# 非小细胞肺癌*EGFR*基因靶向治疗研究进展

**DOI:** 10.3779/j.issn.1009-3419.2017.01.09

**Published:** 2017-01-20

**Authors:** 卉 张, 树才 张

**Affiliations:** 北京，首都医科大学附属北京胸科医院/北京市结核病胸部肿瘤研究所肿瘤内科 Department of Medical Oncology, Beijing Chest Hospital, Capital Medical University/Beijing Tuberculosis and Thoracic Tumor Research Institute, Beijing 101149, China

**Keywords:** 肺肿瘤, 表皮生长因子受体, 突变, 靶向治疗, Lung neoplasms, Epidermal growth factor receptor, Mutation, Targeted therapy

## Abstract

人表皮生长因子受体酪氨酸激酶抑制剂（epidermal growth factor receptor tyrosine kinase inhibitors, EGFR-TKIs）给*EGFR*基因敏感突变患者带来巨大的临床获益。随着临床和基础研究的不断深入，EGFR-TKI已经越来越受到关注。本综述旨在将2016年EGFR-TKI药物研究进展进行概述。

## Abstract (en)

Epidermal growth factor receptor tyrosine kinase inhibitors (EGFR-TKIs) have brought great clinical benefit to patients with EGFR-sensitive mutations. With the deepening of clinical and basic research, EGFR-TKIs have received more and more attention. In this review, we summarize the latest research development about EGFR-targeted drugs in 2016.

表皮生长因子受体（epidermal growth factor receptor, *EGFR*）基因是非小细胞肺癌（non-small cell lung cancer, NSCLC）中最常见的驱动基因之一。PIONEER研究^[1]^显示51.4%未经选择的亚裔晚期肺腺癌患者伴有*EGFR*敏感突变，在不吸烟腺癌患者中高达60%。近十年来，以吉非替尼、厄洛替尼和埃克替尼为代表的第一代表皮生长因子受体酪氨酸激酶抑制剂（epidermal growth factor receptor tyrosine kinase inhibitors, EGFR-TKIs）已成为*EGFR*突变型晚期NSCLC不可或缺的治疗手段。多项前瞻性临床研究^[2-10]^证实一代EGFR-TKI一线治疗*EGFR*突变的晚期NSCLC在客观缓解率（objective response rate, ORR）和无疾病进展时间（progression free survival, PFS）方面均显著优于传统含铂两药联合方案，充分奠定了EGFR-TKI在*EGFR*敏感突变阳性患者中的一线治疗地位。*EGFR*基因突变一直是近年来研究的热点。现将2016年研究进展综述如下。

## 1 第一代EGFR-TKI药物

EGFR-TKI第一代药物主要包含了吉非替尼、厄洛替尼和埃克替尼，它们的研究与广泛应用大大提高了肺癌*EGFR*基因突变患者的总生存期。最新CONⅥNCE研究^[11]^公布，埃克替尼对照培美曲塞联合顺铂方案一线治疗285例*EGFR* 19/21突变的Ⅲb期/Ⅳ期肺腺癌患者，ORR显著高于化疗组（64.8% *vs* 33.8%, *P*＜0.001）；中位PFS达到296 d，也显著长于化疗组（219 d, HR=0.67, *P*=0.008）。并且埃克替尼组与化疗组相比，不良事件、3级以上药物相关不良反应发生率均显著更低。2016年世界肺癌大会（World Conference on Lung Cancer, WCLC）上公布的一项对比埃克替尼与全脑放疗联合化疗应用于*EGFR*突变伴脑转移NSCLC患者的Ⅲ期临床研究（BRAIN, CTONG 1201）^[12]^结果显示，埃克替尼显著提高了患者的颅内无进展生存时间（10.0个月*vs* 4.8个月，*P*=0.014），体现了优越的客观缓解率（67.1% *vs* 40.9%, *P*＜0.001）和疾病控制率（55.0% *vs* 11.1%, *P*＜0.001）。该项研究结果充分证明了对于*EGFR*突变的NSCLC脑转移患者，一线应用埃克替尼是推荐的首选治疗方案。然而，不论一代EGFR-TKI药物已经显示出怎样优越的疗效，耐药的出现仍不可避免，绝大部分患者EGFR-TKI一线治疗6个月-12个月^[1]^后即出现疾病进展。耐药后如何治疗，是目前临床研究关注的热点。2016年欧洲肿瘤内科学会（European Society for Medical Oncology, ESMO）上报道的IMPRESS研究^[13]^在一线吉非替尼治疗进展后的患者中加用培美曲塞联合顺铂方案化疗，对照单纯化疗的患者，结果发现无论是ORR还是PFS均无明显差异（*P*=0.760; *P*=0.273），中位生存时间（overall survival, OS）吉非替尼联合化疗组低于单纯化疗组（13.4个月*vs* 19.5个月，HR=1.44，*P*=0.016）。这一结果告诫我们不要在患者出现影像学进展后继续给予一代EGFR-TKI药物治疗。在按血浆中T790M突变状态进行的亚组分析中发现，T790M突变阳性患者联合化疗组低于单纯化疗组（10.8个月*vs* 14.1个月，HR=1.49），提示疗效可能取决于T790M突变状态，T790M突变阳性患者应接受第三代EGFR-TKI药物治疗。

## 2 EGFR-TKI耐药的分子机制

Jackman等^[14]^对获得耐药进行了明确定义：①既往接受过单药EGFR-TKI治疗；②符合以下标准之一：存在*EGFR*基因敏感突变（如G719X、19del、L858R、L561Q）；一线使用EGFR-TKI有临床获益，包括完全缓解（complete response, CR）、部分缓解（partial response, PR）或超过6个月的疾病稳定（stable disease, SD）；③持续接受EGFR-TKI治疗至少1个月后疾病进展；停用EGFR-TKI及开始新的治疗前未接受其他全身治疗。目前已有证据^[15]^显示其机制主要涉及以下方面：*EGFR*二次突变（如T790M突变的出现）；*Met*基因扩增；*HER2*基因扩增；EGFR下游信号分子活化；旁路激活；表型转化等。T790M突变是EGFR-TKI获得性耐药最常见的机制，其他二次突变如D761Y和L747突变也见报道，但发生率远远低于T790M突变。应用EGFR-TKI治疗的NSCLC患者出现获得性耐药的患者中50%可检测到T790M突变^[16]^。

## 3 第三代EGFR-TKI药物

### 3.1 奥希替尼Osimertinib（AZD9291）

奥希替尼是新一代不可逆性EGFR-TKI，对*EGFR*敏感突变和T790M耐药突变均有更好的作用。2016年发表的Ⅱ期临床AURA2试验最新数据^[17]^显示，应用奥希替尼80 mg *qd*治疗EGFR-TKI治疗后进展T790M突变阳性的210例Ⅲb期/Ⅳ期NSCLC，截至2015年11月，140例/199例（70%）达到客观缓解，其中CR 6例，PR 134例。最常见的3级/4级不良事件为肺栓塞7例（3%），心电图上的QT延长5例（2%），中性粒细胞计数下降4例（2%）。52例（25%）患者发生严重不良事件，研究者评估11例（5%）可能与奥希替尼治疗相关，唯一治疗相关致死事件为间质性肺病。由于奥希替尼用于T790M阳性的NSCLC治疗的疗效非常确切。美国食品和药物管理局（Food and Drug Administration, FDA）在2015年11月13日以加速批准的方式提前3个月批准了奥希替尼用于治疗EGFR-TKI治疗中或治疗后出现进展并伴有*EGFR* T790M阳性突变的NSCLC患者。2016年WCLC公布了奥希替尼对比铂类联合培美曲塞化疗治疗经一线EGFR-TKI治疗后进展的NSCLC的随机Ⅲ期临床研究结果。该项研究（AURA3）^[18]^共纳入419例患者，2:1随机分配至治疗组（奥希替尼80 mg/d，*n*=279）和铂类联合培美曲塞化疗组（*n*=140）。入组患者均为组织活检确认T790M突变，一线EGFR-TKI治疗后影像学进展。主要研究终点为PFS。结果显示奥希替尼组较培美曲塞化疗组PFS明显改善（HR=0.30），分别为10.1个月*vs* 4.4个月，ORR也明显优于化疗组，分别为71% *vs* 31%（OR=5.39），中位缓解持续时间奥希替尼组达到9.7个月，而化疗组仅为4.1个月。奥希替尼最常见的相关不良反应为腹泻（29%）和皮疹（28%），≥3级治疗相关不良事件发生率为6%（*n*=16），明显低于化疗组34%（*n*=46）。该项研究结果更充分证实了奥希替尼治疗一线EGFR-TKI治疗失败且T790M突变阳性的晚期NSCLC患者，疗效优于化疗，且安全性可靠，据此为患者的临床治疗建立了新的标准。该项研究结果已同步发表在*The New England Journal of Medicine*杂志上。而关于奥希替尼在T790M突变的东亚晚期NSCLC患者的Ⅱ期开放性研究（AURA17）结果也在2016年WCLC大会上有初步的报道^[19]^。截至2016年3月4日，共入组171例患者，其中166例疗效可评价，中位治疗时间5.6个月。经独立评审中心（BICR）确认的ORR和疾病控制率（disease control rate, DCR）分别为60%和88%，中位DCR和PFS还未成熟，最常见的AE发生率以及≥3级以上AE发生率分别为腹泻（29%, 0）、皮疹和痤疮（20%, 0）、皮肤干燥（17%, 1%），3例患者报道有间质性肺病。这一结果与全球研究结果相似，是未来奥希替尼在中国注册临床的重要试验。

既往研究结果中奥希替尼在脑转移的治疗中也显露出良好的疗效。2016年ASCO口头报告的BLOOM研究^[20]^结果显示奥希替尼在经治的*EGFR*突变的软脑膜转移患者中的疗效是令人振奋的。该研究纳入EGFR-TKI治疗耐药的并脑脊液细胞学确诊为脑膜转移的21例NSCLC患者，全部给予奥希替尼160 mg *qd*治疗，结果截至2016年3月10日，15例患者还在接受治疗，其中7例患者治疗时间超过9个月，7例患者（33%）达到已确认的影像学好转；9例患者（43%）达到已确认的颅内SD，另外有2例未确认的颅内SD；有2例患者退出研究。5例患者有确认的神经功能提升。没有药物相关的AE导致药物中断或减量。这一研究结果提示奥希替尼在治疗中枢神经系统转移具有显著的疗效。

### 3.2 Rociletinib（CO-1686）

Rociletinib（CO-1686）是另一个第三代EGFR-TKI药物，与奥希替尼的研发一直处于竞争状态。2015年4月*The New England Journal of Medicine*发表TIGER-X^[21]^的初步研究结果显示，未经确认的T790M阳性500 mg组CO-1686治疗的ORR为60%，625 mg组ORR为54%。但之后的再次疗效确认显示500 mg组ORR为28%，625 mg组ORR为34%，疗效稳定性欠佳。2016年ASCO报道^[22]^的研究结果中CO-1686所有剂量组汇总研究者的经确认的ORR为33.9%，且无论由血液、组织、尿液哪种标本类型检测出的T790M突变阳性患者，接受CO-1686治疗，ORR缓解率相似，分别为32.1%、33.9%和36.7%。但在安全性方面，入组的548例患者中有35.2%的患者出现3级以上高血糖，10.2%的患者出现3级以上QT间期延长。基于这些研究结果，美国FDA抗癌药专家委员会反对CO-1686的上市申请。因此CO-1686的临床研发已经中断。但在该项研究中，对T790M突变的检测经验仍值得借鉴。T790M血液、组织和尿液检测可相互补充，每种标本检测均可发现其他标本未检测出的病例，而且无论哪种标本检测出的T790M阳性患者，接受CO-1686治疗，缓解率均相似，说明血液和尿液*EGFR*突变检测应被视为可行的方法，尤其是在肿瘤组织无法获得的情况下。

### 3.3 Olmutinib（BI1482694/HM61713）

HM61713也是针对T790M突变的第三代EGFR-TKI。已于2016年5月17日在韩国获得批准上市，用于既往接受过TKI治疗的局部晚期或转移性*EGFR* T790M突变NSCLC患者。Ⅰ期/Ⅱ期HM-EMSI-101研究中^[23]^，HM61713 800 mg/d显示了显著的临床疗效和良好的安全性，治疗ORR达到62%，DCR达到91%；在出现缓解的76例患者中，有32例在数据采集截止日（2015年6月30日）仍保持有效。治疗相关的主要不良反应包括腹泻、恶心、皮疹、皮肤瘙痒。确认的独立评审ORR为54%，中位DCR为8.3个月，最常见的不良反应均为轻度至中度，多为胃肠道和皮肤反应。该药目前正在进行全球多中心Ⅱ期临床研究（NCT02485652），用于进一步评估HM61713在T790M阳性NSCLC患者的疗效与安全性。

### 3.4 艾维替尼（AC0010）

艾维替尼是在我国自主研发、具有创新性的第三代EGFR-TKI，临床前期数据显示一线TKI耐药后可起作用，目前正进行临床研究。2016年ESMO大会口头报告了其初步结果^[24]^，是艾维替尼首次在人体进行的临床试验。一代TKI治疗耐药后患者接受艾维替尼的爬坡试验，剂量从50 mg *qd*到600 mg *qd*。初步研究结果显示总体ORR为38.2%，对T790M突变阳性的患者显示出显著的抗肿瘤活性，每日剂量350 mg和600 mg，ORR为55.6%（20/36），每日剂量175 mg和300 mg，ORR为62%（13/21）。主要不良反应有腹泻（44%）、皮疹（20%）、瘙痒（16%），腹泻和皮疹的频率增加成剂量依赖性。250 mg *bid*和300 mg *bid*显示出更好的抗肿瘤活性，是未来Ⅱ期临床研究的推荐剂量，剂量扩展研究正在进行中。

### 3.5 其他第三代EGFR-TKI药物：ASP8273、EGF816

目前ASP8273和EGF816都只完成了Ⅰ期临床研究^[25, 26]^。2016年ASCO上报告110例接受过EGFR-TKI治疗的*EGFR*突变阳性的患者接受了ASP8273不同剂量的治疗，其中300 mg亚组（63例）PFS 6.0个月，ORR 31%，表现出良好安全性和耐受性。ASP8273 300 mg/d组的大样本（*n*=600）的随机Ⅲ期临床研究（SOLAR），对比厄洛替尼/吉非替尼治疗*EGFR*突变晚期NSCLC（NCT02588261）的有效性及安全性/耐受性正在进行中。EGF816的Ⅰ期临床研究中所有剂量组ORR为46.9%，中位PFS为9.7个月，最常见药物相关不良反应是皮疹、腹泻，多数不良反应较轻，耐受性良好。关于进一步观察EGF816在NSCLC患者中的抗肿瘤活性的Ⅱ期临床研究即将展开。

## 4 Osimertinib（AZD3759）

AZD3759是一种口服的可穿透血脑屏障的可逆性EGFR突变抑制剂，主要为提高脑脊液及血液中的游离药物浓度。AZD3759在临床前期脑-软脑膜转移患者中抗肿瘤活性显著，脑脊液/血浆比为1:1。Ⅰ期临床研究^[27]^共入组29例患者，均为亚洲人，分别有7例和2例患者在血浆和脑脊液检测出T790M突变。药物相关不良反应与其他EGFR-TKI相符，主要是皮疹和腹泻，没有发现药物相关的中枢神经系统不良反应。Ⅱ期临床试验推荐剂量为200 mg *bid*，21例脑转移患者在入选前接受过多线治疗，且颅内及颅外病灶在入组时已出现进展，13例曾接受过短暂的EGFT-TKI治疗。接受剂量≥50 mg *bid*治疗的11例患者观察到颅内肿瘤的缩小，其中3例PR患者疗效确认，22例颅外病灶可测量的患者中，8例患者肿瘤缩小。这些结果给终末期脑转移患者带来了新的希望，期待更大样本量的Ⅲ期临床研究。

## 5 第四代EGFR抑制剂EAI045

位于EGFR酪氨酸激酶结构域的C797S突变被认为是针对T790M突变的第三代EGFR不可逆抑制剂的主要耐药机制。EAI045是目前为止第一个针对T790M及C797S突变设计的变构抑制剂。研究^[28]^证实，EAI045对于具有二聚体缺陷的*EGFR*突变有更强的活性。当与西妥昔单抗（可以阻止EGF与配体结合从而阻断EGFR二聚体化的单抗）联合，EAI045显著抑制了具有L858R/T790M突变的Ba/F3细胞系的增殖。在带有L858R/T790M突变的肺癌转基因小鼠模型中，分别单独或与西妥昔单抗联合测试EAI045的效果，发现联合处理的小鼠中肿瘤显著消退，而单独使用未见有效。在带有L858R/T790M/C797S突变的细胞系和小鼠模型中也有类似的现象，这些结果可以证明EAI045能够克服T790M/C797S突变导致的获得性耐药，但只有在与西妥昔单抗联用才有效。在NSCLC患者的治疗有效性与安全性尚需临床研究验证。

## 6 展望

针对T790M突变的第三代EGFR-TKI临床研究正如火如荼，目前只有奥希替尼获得美国FDA批准上市，用于治疗EGFR-TKI治疗中或治疗后出现进展并伴有*EGFR* T790M阳性突变的NSCLC患者。但2016年众多的研究结果让我们看到了彻底克服T790M突变的治疗希望。但是并不是所有一代EGFR-TKI耐药都是由T790M产生，制定耐药后的治疗策略，关键要看耐药的机制是什么，只有机制搞清楚，才有可能针对性地解决。因此在制订第一代EGFR-TKI耐药后治疗策略之前，最好能再次对驱动基因突变情况加以检测，才能做到真正的个体化精准治疗。

## References

[1] Shi Y, Li J, Zhang S (2015). Molecular epidemiology of *EGFR* mutations in asian patients with advanced non-small-cell lung cancer of adenocarcinoma histology-Mainland China Subset Analysis of the PIONEER study. PLoS One.

[2] Mok TS, Wu YL, Thongrasert S (2009). Gefitinib or carboplatin-paclitaxel in pulmonary adenocarcionoma. N Engl J Med.

[3] Mitsudomi T, Morita S, Yatabe Y (2010). West Japan Oncology Group. Gefitinib versus cisplatin plus docetaxel in patients with non-small-cell lung cancer harbouring mutation of the epidermal growth factor receptor (WJTOG3405): an open label, randomized phase 3 trial. Lancet Oncol.

[4] Maemondo M, Inoue A, Kobayashi K (2010). North-East Japan Study Group. Gefitinib or chemotherapy for non-small-cell lung cancer with mutated *EGFR*. N Engl J Med.

[5] Zhou C, Wu YL, Chen G (2011). Erlotinib versus chemotherapy as first-line treatment for patients with advanced *EGFR* mutation-positive non-small-cell lung cancer (OPTIMAL, CTONG-0802): a multicentre, open-label, randomised, phase 3 study. Lancet Oncol.

[6] Han JY, Park K, Kim SW (2012). First-SIGNAL: first-line single-agent iressa versus gemcitabine and cisplatin trial in never-smokers with adenocarcinoma of the lung. J Clin Oncol.

[7] Rosell R, Carcereny E, Gervais R (2012). Spanish Lung Cancer Group in collaboration with Groupe Francais de Pneumo-Cancerologie and Associazione Italiana Oncologis Toracica. Erlotinib versus standard chemotherapy as first-line treatment for European patients with advanced *EGFR* mutation-positive non-small-cell lung cancer (EURTAC): a multicentre, open-label, randomised phase 3 trial. Lancet Oncol.

[8] Seauist LV, Yang JC, Yamamoto N (2013). Phase Ⅲ study of afatinib or cisplatin plus pemetrexed in patients with metastatic lung adenocarcinoma with *EGFR* mutations. J Clin Oncol.

[9] Wu YL, Zhou C, Hu CP (2014). Afatinib versus cisplatin plus gemcitabine for first-line treatment of Asian patients with advanced non-small-cell lung cancer harbouring *EGFR* mutations (LUX-Lung 6): an open-label, randomised phase 3 trial. Lancet Oncol.

[10] Yang JC, Wu YL, Schuler M (2015). Afatinib versus cisplatin-based chemotherapy for *EGFR* mutation-positive lung adenocarcinoma (LUX-Lung 3 and LUX-Lung 6): analysis of overall survival data from two randomised, phase 3 trails. Lancet Oncol.

[11] Shi Y, Wang L, Han B (2016). First-line icotinib versus cisplatine/pemetrexed plus pemetrexed maintenance therapy in lung adenocarcinoma patients with sensitizing EGFR mutation (CONVINCE). J Clin Oncol.

[12] Wu YL, Yang JJ, Zhou CC (2017). BRAIN: A phase Ⅲ trial comparing WBI and chemotherapy with icotinib in NSCLC with brain metastases harboring *EGFR* Mutations (CTONG 1201). J Thoracic Oncol.

[13] Soria J, Kim S, Wu Y (2016). Gefitinib/chemotherapy *vs* chemotherapy in *EGFR* mutation-positive NSCLC after progression on 1^st^ line gefitinib (IMPRESS study): Final overall survival (OS) analysis. Ann Oncol.

[14] Jackman D, Pao W, Riely GJ (2010). Clinical definition of acquired resistance to epidermal growth factor receptor tyrosine kinase inhibitors in non-small-cell lung cancer. J Clin Oncol.

[15] Morgillo F, Della Corte CM, Fasano M (2016). Mechanisms of resistance to EGFR-targeted drugs: lung cancer. ESMO Open.

[b16] 16Wang S, Song Y, Yan F, *et al*. Mechanisms of resistance to third-generation EGFR tyrosine kinase inhibitors. Front Med, 2016 Oct 21. [Epub ahead of print]

[17] Goss G, Tsai CM, Shepherd FA (2016). Osimertinib for pretreated EGFR Thr790Met-positive advanced non-small-cell lung cancer (AURA2): a multicentre, open-label, single-arm, phase 2 study. Lancet oncol.

[18] Papadimitrakoupoulou V, Wu YL, Ahn MJ (2017). Randomised phase Ⅲ study of osimertinib *vs* platinum-pemetrexed for *EGFR* T790M-positive advanced NSCLC (AURA3). J Thoracic Oncol.

[19] Ahn MJ, Cantarini M, Chen Y (2017). Osimertinib in Asia-Pacific patients with T790M mutation-positive advanced NSCLC: open-label Ⅱ study results. J Thoracic Oncol.

[20] Yang CH, Kim DW, Kim SW (2016). Osimertinib activity in patients (pts) with leptomeningeal (LM) disease from non-small cell lung cancer (NSCLC): Updated results from BLOOM, a phase Ⅰ study. J Clin Oncol.

[21] Sequist LV, Soria JC, Goldman JW (2015). Rociletinib in *EGFR*-mutated non-small-cell lung cancer. N Engl J Med.

[22] Wakelee HA, Gadgeel SM, Goldman JW (2016). Epidermal growth factor receptor (EGFR) genotyping of matched urine, plasma and tumor tissue from non-small cell lung cancer (NSCLC) patients (pts) treated with rociletinib. J Clin Oncol.

[23] Park K, Lee JS, Lee KH (2016). BI 1482694 (HM61713), an *EGFR* mutant-specific inhibitor, in T790M + NSCLC: Efficacy and safety at the RP2D. J Clin Oncol.

[24] Zhang L, Zhao H, Hu B (2016). First-in-human study of AC0010, a novel irreversible, mutant-selective EGFR inhibitor in patients with 1^st^ generation EGFR TKI-resistant non-small cell lung cancer (NSCLC). Annals of Oncology.

[25] Yu HA, Spira AI, Horn L (2016). Antitumor activity of ASP8273 300 mg in subjects with *EGFR* mutation-positive non-small cell lung cancer: interim results from an ongoing phase 1 study. J Clin Oncol.

[26] Tan SW, Yang CH, Leighl NB (2016). Updated results of a phase 1 study of EGF816, a third-generation, mutant-selective EGFR tyrosine kinase inhibitor (TKI), in advanced non-small cell lung cancer (NSCLC) harboring T790M. J Clin Oncol.

[27] Ahn MJ, Kim DW, Kim TM (2016). Phase Ⅰ study of AZD3759, a CNS penetrable EGFR inhibitor, for the treatment of non-small-cell lung cancer (NSCLC) with brain metastasis (BM) and leptomeningeal metastasis (LM). J Clin Oncol.

[28] Wang S, Song Y, Liu D (2017). EAI045: The fourth-generation EGFR inhibitor overcoming T790M and C797S resistance. Cancer Lett.

